# Mesonephric-like Adenocarcinoma of the Uterine Corpus: Comprehensive Immunohistochemical Analyses Using Markers for Mesonephric, Endometrioid and Serous Tumors

**DOI:** 10.3390/diagnostics11112042

**Published:** 2021-11-04

**Authors:** Hyunjin Kim, Kiyong Na, Go Eun Bae, Hyun-Soo Kim

**Affiliations:** 1 Department of Pathology and Translational Genomics, Samsung Medical Center, Sungkyunkwan University School of Medicine, Seoul 06351, Korea; hkim.pathol@gmail.com; 2 Department of Pathology, Kyung Hee University Hospital, Kyung Hee University College of Medicine, Seoul 02447, Korea; raripapa@gmail.com; 3 Department of Pathology, Chungnam National University School of Medicine, Daejeon 35015, Korea

**Keywords:** uterus, mesonephric-like adenocarcinoma, endometrium, endometrioid carcinoma, serous carcinoma, immunohistochemistry

## Abstract

Mesonephric-like adenocarcinoma (MLA) of the uterine corpus is a rare but distinct malignant tumor of the female genital tract, demonstrating a characteristic morphology and unique immunohistochemical profiles and molecular alterations. We conducted immunohistochemical staining (IHC) to make precise differential diagnoses of uterine MLAs from common histological subtypes of endometrial carcinomas. We collected 25 uterine MLAs and performed IHC for GATA3, TTF1, CD10, ER, PR, p16, p53, and HER2. Seventeen cases (68.0%) showed at least moderate nuclear GATA3 immunoreactivity in ≥25% of tumor cells. Most cases expressed TTF1 (17/21, 81.0%) and CD10 (luminal; 17/21, 81.0%). Heterogeneous TTF1 expression was noted in 12 cases. An inverse pattern of GATA3 and TTF1 staining was observed in eight cases (32.0%). Three cases (12.0%) showed moderate-to-strong ER expression in ≥25% of tumor cells, and two cases (8.0%) showed moderate-to-strong PR expression in ≥5% of tumor cells. These hormone receptor-positive MLAs varied in intensity and proportion of GATA3 staining. None of the 25 cases exhibited either diffuse and strong p16 expression or aberrant p53 expression. Five cases (20.0%) showed equivocal HER2 immunoreactivity (score 2+), but *HER2* FISH confirmed that none of them exhibited *HER2* gene amplification. In summary, a small subset of uterine MLAs displayed atypical IHC results: focal but strong expression of ER or PR, the complete absence of GATA3 immunoreactivity, the concurrent expression of mesonephric and hormone receptors, and the inverse pattern of GATA3 and TTF1 staining. These unusual immunophenotypes may complicate the differential diagnosis of MLA. Moreover, pathologists should be encouraged to interpret the IHC results cautiously.

## 1. Introduction

Mesonephric adenocarcinoma (MA) is a rare malignant tumor of the female genital tract that is thought to derive from the embryonal remnant of the mesonephric tubules and ducts [1,2]. It comprises less than 1% of all gynecological malignancies [3]. MAs typically arise in the lateral wall of the uterine cervix or vagina; however, MAs of the uterine corpus and adnexa have also been reported [2,4]. MA of the upper female genital tract has been referred to as mesonephric-like adenocarcinoma (MLA), as its relationship with the mesonephric remnant has not been firmly established [3,4]. MLA of the uterine corpus is only recently described: its first mention in the World Health Organization (WHO) Classification of Female Genital Tumors was in 2020 [5]. However, uterine MLA has already been vigorously studied due to its aggressive behavior, poor prognosis, and the difficulty of differentiating it from two common histological subtypes of endometrial carcinoma: endometrioid carcinoma (EC) and serous carcinoma (SC) [5,6,7,8].

Currently, there are no standardized or robust immunohistochemical staining (IHC) panels for the diagnosis of uterine MLA. Although the 2020 WHO Classification documents ‘desirable’ criteria as positive expression of GATA-binding protein 3 (GATA3) and thyroid transcription factor (TTF1), with negative expression of hormone receptors [5], these are not sufficient for the reliable diagnosis of this rare tumor; the development of more effective diagnostic criteria is critical. Therefore, we used IHC to investigate the immunophenotypes of uterine MLA, with a primary focus on the conventional markers used for endometrial EC and SC, including estrogen receptor (ER), progesterone receptor (PR), p16, p53, human epidermal growth factor receptor 2 (HER2), and the mesonephric markers GATA3, TTF1, and CD10.

## 2. Materials and Methods

### 2.1. Case Selection

**Figure 1:**
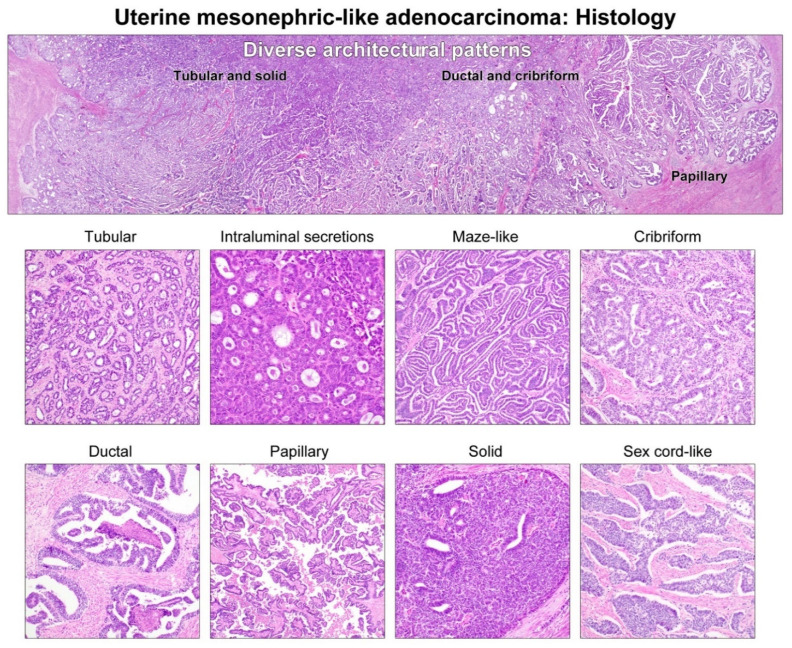
Histological features of uterine MLA: architectural diversity. The classic morphological pattern of uterine MLA is tubular, with small, compactly aggregated tubules containing densely eosinophilic intraluminal secretions. Additional morphological patterns are maze-like, cribriform, ductal (endometrioid), papillary, solid, sex cord-like, etc. Such architectural diversity poses a challenge in the differential diagnosis of uterine MLA and the more common histological subtypes of endometrial carcinomas, including EC and SC.

Two experienced gynecological pathologists (H.K. and H.-S.K.) reviewed all available hematoxylin and eosin-stained slides. Twenty-five cases of uterine MLA were diagnosed by characteristic histological features (Figure 1), including the presence of (1) a tubular growth pattern with small, closely packed, back-to-back tubules lined by cuboidal cells and containing eosinophilic intraluminal secretions, and (2) diverse architectural patterns (e.g., ductal, papillary, maze-like, sieve-like, solid, spindle, retiform, sex cord-like, comedonecrosis-like, glomeruloid, etc.) [1,5,6,7,8,9,10,11,12,13,14,15]. The most representative block of each case was selected for IHC and next-generation sequencing (NGS). MLAs found to exhibit equivocal HER2 immunoreactivity (IHC score 2+) were to be subsequently analyzed with *HER2* fluorescence in situ hybridization (FISH). Twenty-one patients (cases 1–5 and 7–22) underwent surgical staging in our institution, whereas four cases (cases 6 and 23–25) were retrieved from the consultation file of one of the authors (H.-S.K.).

### 2.2. IHC

Briefly, 4-µm-thick, formalin-fixed, paraffin-embedded (FFPE) tissue slices were deparaffinized and rehydrated using a xylene and alcohol solution. IHC was performed using a BOND-MAX automated immunostainer (Leica Biosystems, Buffalo Grove, IL, USA) [1,8,9,16,17,18,19,20,21]. After antigen retrieval, the slices were incubated with the primary antibodies (Table 1). After chromogenic visualization, the slides were counterstained with hematoxylin. Appropriate controls were stained concurrently. Positive controls were luminal A invasive mammary carcinoma for GATA3, ER, and PR, papillary thyroid carcinoma for TTF1, normal proliferative-phase endometrial stroma for CD10, ovarian high-grade serous carcinoma for p16 and p53, and HER2-enriched invasive mammary carcinoma for HER2. Negative controls were prepared by substituting non-immune serum for primary antibodies, which resulted in undetectable staining.

### 2.3. IHC Interpretation

Each immunostained slide was scored by pathologists (H.K. and H.-S.K.) [8,9,10,16,17,18,19,22,23,24,25,26,27]. The staining intensities of GATA3, ER, PR, TTF1, and CD10 were designated as either negative, weak, moderate, or strong, and the staining proportions were determined in increments of 5% across a 0–100% range. Histo score (H-score), a summation of the proportion of area stained at each intensity level multiplied by the weighted staining intensity (e.g., 0, negative; 1, weak; 2, moderate; 3, strong), was generated [28,29]. A p53 IHC pattern was considered aberrant (i.e., mutant) when any one of the following features was observed: diffuse and strong nuclear immunoreactivity in ≥75% of tumor cells (i.e., overexpression); no nuclear immunoreactivity in any tumor cell (i.e., complete absence); or unequivocal cytoplasmic staining (i.e., cytoplasmic pattern). Immunostained slides exhibiting a variable proportion of tumor cell nuclei expressing p53 with mild-to-moderate intensity were considered wild-type [30]. The p16 IHC pattern was considered diffuse and strong when p16 was expressed in the nuclei with continuous and strong staining regardless of any cytoplasmic reactions. All other p16 IHC patterns, including focal nuclear staining and wispy, blob-like, puddled, and scattered cytoplasmic staining, were interpreted as patchy [8,10,24,25,26]. 

HER2 IHC was scored with strict adherence to the recommendations of the International Society of Gynecological Pathologists (ISGyP) Companion Society Session [31]. HER2 was considered positive with either an IHC score of 3+ or with an IHC score of 2+ in combination with confirmation of *HER2* gene amplification by fluorescence in situ hybridization (FISH). An HER2 IHC score of 3+ was assigned when >30% of tumor cells demonstrated strong complete or lateral/basolateral membranous immunoreactivity. An HER2 score of 2+ was assigned when (1) ≤30% of tumor cells exhibited strong complete or lateral/basolateral membranous staining, or (2) when weak-to-moderate staining was observed in ≥10% of tumor cells. FISH was performed only on tumors with an IHC score of 2+ on a large tumor area (≤1 cm^2^) in direct correlation with the HER2-stained slide.

### 2.4. FISH

Per the recommendations of the ISGyP [31], *HER2* FISH was performed in the five MLAs that received HER2 IHC scores of 2+. For FISH, two-µm-thick FFPE slices were deparaffinized and pretreated [32,33]. After protease treatment and fixation, the slides were incubated with the DNA probe (PathVysion HER-2 DNA Probe Kit, Abbott Laboratories, Abbott Park, IL, USA) at 73 °C for 5 min and at 37 °C for 20 h. The slides were counterstained with 20 µL of 4′,6-diamidino-2-phenylindole II, covered with coverslips, and visualized with an automatic fluorescence imaging system (BioView Duet, BioView Inc., Billerica, MA, USA). *HER2* was considered amplification with a *HER2*/centrosomal area of chromosome 17 (CEP17) ratio of ≥2.0 [31].

### 2.5. Statistical Analysis

Fisher’s exact test was performed to examine the differences between discrete variables. Univariate survival analysis with a Kaplan–Meier plot was performed to examine the prognostic significance with respect to the disease-free survival (DFS). All statistical analyses were performed using IBM SPSS Statistics for Windows, version 23.0 (IBM Corp., Armonk, NY, USA). Statistical significance was defined as *p* < 0.05.

### 2.6. NGS

Five-micrometer-thick FFPE tissue slides were deparaffinized and hydrated through graded alcohols to water. The slides were manually microdissected using a scalpel point dipped in ethanol. The scraped material was washed in phosphate-buffered saline and digested in proteinase K overnight at 56 °C in Buffer ATL (Qiagen, Hilden, Germany). DNA and RNA were then isolated using a QIAamp DSP DNA FFPE Tissue Kit (Qiagen) [34]. A Qubit 4 Fluorometer (Thermo Fisher Scientific, Waltham, MA, USA) was used for sample quantitation by means of the highly sensitive and accurate fluorescence-based quantitation assays. A NGS library was prepared using extracted DNA and RNA and the Ion AmpliSeq Library Preparation on the IonChef System protocol (Thermo Fisher Scientific). Sequencing was performed on the IonTorrent S5 XL platform, using the Oncomine Comprehesive Assay v3 (Thermo Fisher Scientific), an amplicon-based, targeted assay that enables the detection of relevant single-nucleotide variants, amplifications, gene fusions, and indels from 161 unique genes, and positive control cell line mixtures (Horizon Discovery, Cambridge, UK). Genomic data were analyzed, and alterations were detected using the IonReporter Software v5.6 (Thermo Fisher Scientific). We also manually reviewed the variant call format file and integrated genomic viewer. Only variants in coding regions, promoter regions, or splice variants were retained.

## 3. Results

### 3.1. Clinicopathological Characteristics

Table 2 summarizes the clinicopathological characteristics of 25 patients with uterine MLA. The mean patient age was 58.8 years (median, 59 years; range, 43–77 years). Seven patients had FIGO stage I disease, two were stage II, 10 were stage III, and six were stage IV. Postoperative follow-up periods ranged from 4 to 60 months. Clinical information related to postoperative locoregional recurrence or distant metastasis was available in all except two patients (cases 7 and 17) whose follow-up periods were less than three months. Eighteen patients (72.0%) developed postoperative local or metastatic recurrences, with a mean DFS of 22.6 months. More than half of the patients (14/25; 56.0%) had lung metastases either at the time of initial diagnosis or after surgery. One patient (case 20) died six months after surgery; all others were alive at the time of writing.

### 3.2. Histological Features and Immunophenotype of Mesonephric-like Carcinosarcoma (MLCS)

**Figure 2:**
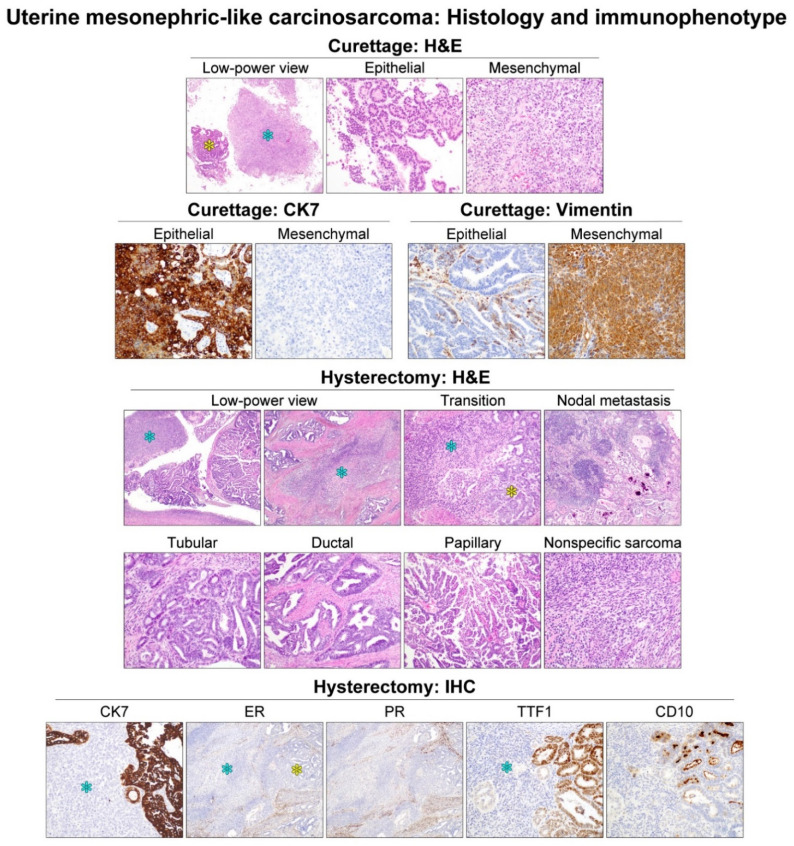
Histological features and immunophenotype of uterine MLCS: representative case (case 11). The endometrial curettage specimen revealed both the epithelial (yellow asterisk) and mesenchymal (blue asterisk) components, compatible with the diagnosis of carcinosarcoma. Immunostaining results further supported the diagnosis because the epithelial component was positive for cytokeratin 7 (CK7) but negative for vimentin, and the opposite in the mesenchymal component. The hysterectomy specimen also revealed two histological components. Several microscopic foci showing a transition between the two components were noted. The epithelial component showed various growth patterns, including tubular, ductal, and papillary architecture, and a typical immunophenotype for MLA, including TTF1 and CD10 positivity accompanied with hormone receptor negativity. These findings were characteristic of uterine MLA. Nodal metastatic tumor consisted exclusively of MLA. The mesenchymal component, morphologically high-grade nonspecific sarcoma, did not react with any hormone receptor or mesonephric marker.

Four cases (cases 6, 11, 18, and 25) were diagnosed as MLCS. Their endometrial curettage and hysterectomy specimens revealed both the epithelial and mesenchymal components, compatible with the diagnosis of MLCS. MLA was an epithelial component in each of the four MLCS cases, occupying approximately 50% (case 6), 80% (case 18), and 90% (cases 11 and 25) of the entire tumor volume. In these cases, the remaining proportion of each tumor comprised high-grade nonspecific sarcoma as a homologous mesenchymal component. A heterologous mesenchymal component was not identified in any of the MLCS cases. In all MLCS cases, we observed several microscopic areas showing a transition between the epithelial and mesenchymal components. In three cases in which the histological examination of the metastatic tumor was available, nodal and distant metastatic tumors consisted exclusively of the epithelial component, i.e., the MLA. Immunohistochemically, the epithelial component was positive for epithelial membrane antigen (EMA) and cytokeratin 7 (CK7), whereas the mesenchymal component was negative or very focally positive for EMA and CK7 but diffusely and strongly positive for vimentin. In two cases, we observed that a small amount of carcinoma cells showed a significant reduction or loss of EMA and CK7 expression at the transitional areas. At the same time, some of the sarcoma cells adjacent to the epithelial component displayed patchy and weak immunoreactivity for the epithelial markers. Representative photomicrographs demonstrating histological features and immunostaining results of uterine MLCS (case 11) are shown in Figure 2.

### 3.3. IHC Results

**Figure 3:**
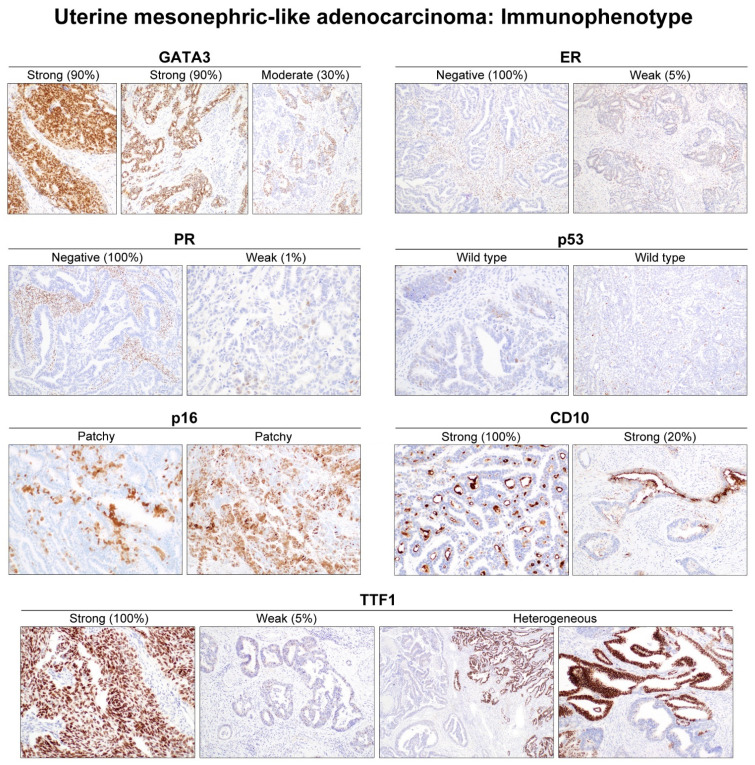
Typical immunophenotype of uterine MLA. In this study, more than two-thirds of uterine MLAs (17/25; 68.0%) were moderately-to-strongly positive for GATA-binding protein 3 in ≥25% of tumor cells. In contrast, most MLAs were negative for hormone receptors, although a few showed focal nuclear immunoreactivity for either estrogen receptor or progesterone receptor, with variable staining intensity. All tumors exhibited scattered p53-positive tumor nuclei with mild-to-moderate staining intensity (i.e., wild-type p53 IHC pattern). Most uterine MLAs demonstrated patchy p16 positivity within the nuclei and cytoplasm. The tubules and ducts showed luminal CD10 immunoreactivity with strong staining intensity. The proportion and intensity of TTF1 staining varied. A heterogeneous pattern of TTF1 expression was observed in 12 (57.1%) of the examined cases. Variable-sized microscopic areas showing a complete lack of TTF1 staining were clearly demarcated from the surrounding ducts or tumor cell clusters that were strongly positive for TTF1.

**Figure 4:**
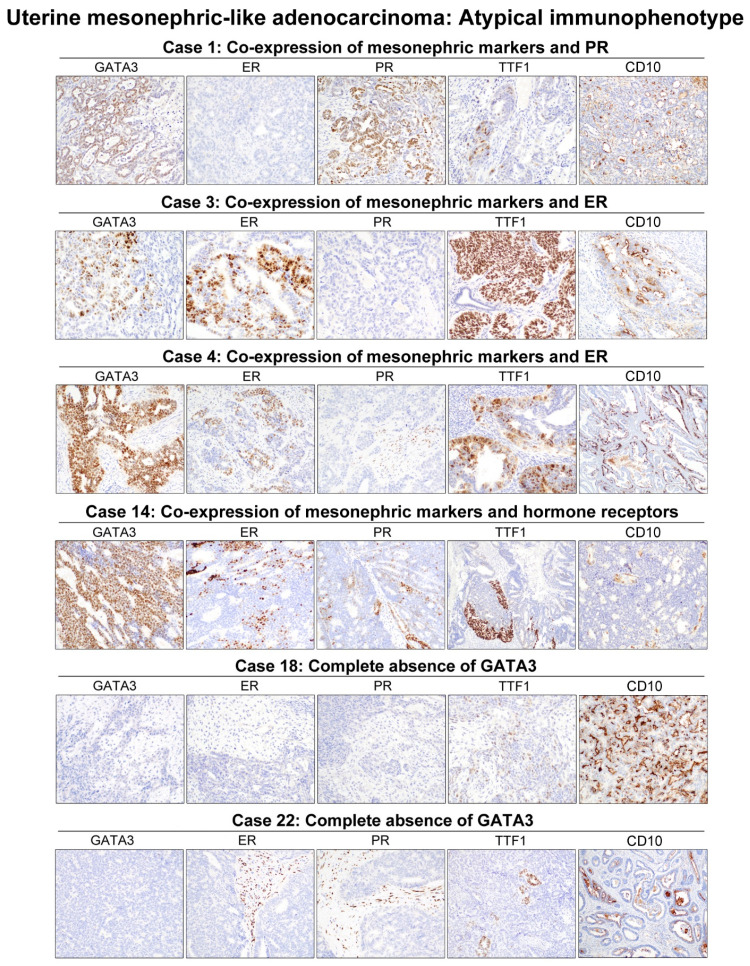
Atypical immunophenotypes. Three tumors (cases 1, 3, and 4) expressed all mesonephric markers and one hormone receptor simultaneously. Concurrent expression of GATA3, TTF1, ER, and PR was noted in one tumor (case 14); in this case, 95% of tumor cells expressed GATA3 with moderate-to-strong intensity whereas ≤25% of tumor cells expressed ER or PR. GATA3 was absent in cases (18 and 22) where TTF1 and CD10 were both positive. The tumor in case 18 was diagnosed as MLCS, in which both the epithelial and mesenchymal components were completely negative for GATA3, ER, PR, and TTF1.

**Figure 5:**
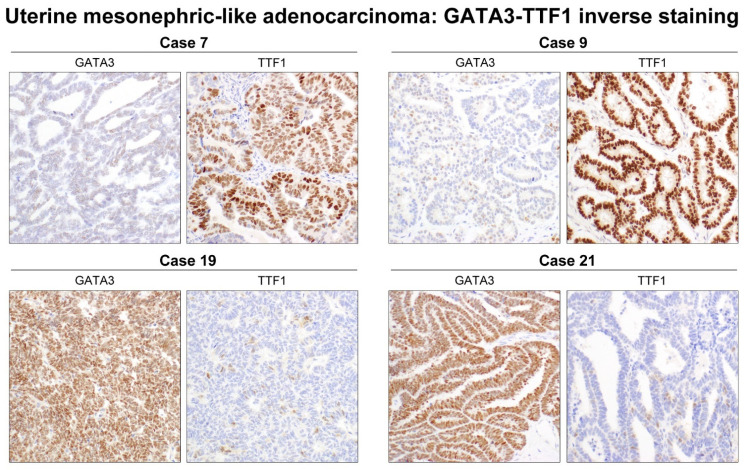
An inverse expression of GATA3 and TTF1. Eight of the 21 cases showed an inverse relationship between GATA3 and TTF1 expression. Four cases showing moderate-to-strong TTF1 immunoreactivity were weakly positive for GATA3 in some scattered tumor cells. In the other four cases, microscopic tumor areas that were strongly positive for GATA3 exhibited faint nuclear TTF1 expression in some tumor cells.

The IHC results of each case are shown in Table 3. Frequencies of positive and negative immunoreactivities for each antibody are summarized in Table 4. Representative photomicrographs demonstrating typical and atypical immunophenotypes of uterine MLA are shown in Figure 3 and Figure 4, respectively. Figure 5 depicts an inverse expression pattern of GATA3 and TTF1.

#### 3.3.1. ER and PR

Although seven tumors showed at least some positivity for at least one hormone receptor, none were predominantly positive in >50% of tumor cells. Specifically, 19 tumors (76.0%) were negative for ER in ≥99% of tumor cells; of these, 17 (68.0%) showed a complete absence of hormone receptor expression. Similarly, 23 tumors (92.0%) were negative for PR; of these, 22 (88.0%) showed a complete absence of hormone receptor expression. Three tumors showed moderate-to-strong ER immunoreactivity in 25–30% of tumor cells, and three tumors showed focal (5–20%) ER expression with weak-to-moderate staining intensity. Two cases showed moderate-to-strong PR expression in 5% and 40% of tumor cells, respectively. In these two cases, the tumor cells expressing either ER or PR were randomly intermingled with the hormone receptor-negative tumor cells. In the four MLCS cases, the mesenchymal component was completely negative for hormone receptors.

#### 3.3.2. GATA3

Seventeen tumors (68.0%) were at least moderately positive for GATA3 in ≥25% of tumor cells. Eight tumors (32.0%) were negative for GATA3 in ≥90% of tumor cells, and two (8.0%) tumors were completely negative for GATA3. In two of the eight GATA3-negative cases (cases 3 and 9), the GATA3-negative tumor cells were positive for at least one hormone receptor. Such GATA3-negative/hormone receptor-positive tumor cells were not limited to any specific architectural pattern, but were randomly dispersed in tubular, ductal, and solid patterns. In the four MLCS cases, the mesenchymal component was completely negative for GATA3.

#### 3.3.3. p16 and p53

All uterine MLAs exhibited a wild-type p53 IHC pattern. Some scattered tumor cells were positive for p53, with variable staining intensity. No tumors displayed diffuse and strong p16 positivity. Twenty-three tumors showed patchy positivity, while two were negative for p16.

#### 3.3.4. HER2

HER2 IHC was scored strictly adhering to the ISGyP Companion Society Session recommendations [31]. Although 20 tumors were negative (0 or 1+) for HER2, five were classified as equivocal (2+). None of the 25 tumors exhibited positive (3+) HER2 expression. Per the recommendations, *HER2* FISH was performed for the five tumors that showed equivocal HER2 immunoreactivity. *HER2* FISH analysis confirmed that none of these tumors exhibited *HER2* gene amplification.

#### 3.3.5. TTF1

TTF1 IHC was examined in 21 cases, all of which demonstrated nuclear TTF1 immunoreactivity. Seventeen of the 21 (81.0%) tumors were diffusely or focally positive for TTF1 with moderate-to-strong staining intensity. The remaining four (19.0%) cases also displayed weak TTF1 expression in 5–10% of the tumor cells. Interestingly, TTF1 immunostaining was geographically heterogeneous in 12 (57.1%) cases. Variable-sized microscopic areas showing a complete lack of TTF1 staining were clearly demarcated from the surrounding tumor cell clusters that were strongly positive for TTF1. We did not identify any significant difference in histological features between TTF1-negative and -positive areas. The mesenchymal component was negative for TTF1 in all MLCS cases.

A recent study by Pors et al. [12] and Euscher et al. [5] reported an inverse expression pattern of GATA3 and TTF1. Consistent with the previous data, our eight (38.1%) cases displayed an inverse relationship between GATA3 and TTF1 staining. Microscopic tumor areas that were positive for TTF1 were focally and weakly positive or negative in four cases (cases 3, 7, 9, and 20), and vice versa in the remaining four cases (cases 1, 14, 19, and 21).

#### 3.3.6. CD10

We observed moderate-to-strong CD10 immunoreactivities along the luminal side of tubules and glands in 17 (81.0%) of the 21 cases examined. Although the staining proportion varied among the cases, all of them exhibited intense CD10 expression in at least 5% of the tumor cells. In some cases, basolateral or circumferential membranous CD10 immunoreactivity was found in a few microscopic areas. The mesenchymal component was negative or focally and weakly positive for CD10.

### 3.4. NGS Results

Twenty-one tumor tissue samples were subjected to NGS. Sixteen (76.2%) cases harbored activating *KRAS* mutations, including p.G12D (8/16), p.G12V (6/16), p.G12C (1/16), and p.G13D (1/16).

### 3.5. Results of Statistical Analysis

Table 5 and Table 6 summarize the results of statistical analyses. The expression status of GATA3, ER, PR, TTF1, and CD10, calculated by H-score, was not significantly associated with initial pathological FIGO stage, postoperative recurrence, or lung metastasis. We did not observe significant differences in DFS according to protein expression status. We could not examine the association between the protein expression status and overall survival since all except one patient were currently alive.

## 4. Discussion

Arriving at a conclusive diagnosis of uterine carcinoma often necessitates testing multiple IHC markers. Since the immunophenotypes of endometrial EC and SC are familiar to most surgical pathologists, they are likely to perform IHC using a panel of antibodies typical for common endometrial carcinomas: ER, PR, p16, and p53. However, the IHC results for these markers in cases of uterine MLA should be interpreted cautiously given the possibility of atypical expression patterns. While many studies have highlighted unique clinicopathological characteristics of uterine MLA [1,5,11,35], none has comprehensively analyzed its immunophenotypes using the conventional markers of endometrial carcinoma. The primary objective of this study was to explore and share the IHC results of uterine MLA, so that this entity is correctly recognized and diagnosed for the appropriate management of the patients. Although the number of MLA cases in our study was relatively small, we believe that our results will help pathologists correctly diagnose uterine MLA.

It has been well known that uterine MLA has a poor prognosis with frequent metastasis, particularly to the lungs in nearly half of the metastatic cases [36]. In the largest multi-institutional study by Pors et al. [7], 58.1% (25/43) of the uterine MLAs were advanced FIGO stage (II or higher) tumors at the time of diagnosis. More than half (58.5%; 24/41) of them developed recurrences, most commonly distant (91.7%; 22/24). The progression-free survival ranged from 18–21 months [5,7], similar to that of high-grade EC and SC, and much lower than low-grade EC. Moreover, even the patients who received surgery for early-stage disease had a tendency to suffer a recurrence and their cancer metastasized frequently. In this regard, previous studies have suggested that uterine MLCs should not be graded but should all be considered high-grade, as SCs and clear cell carcinomas are regarded [4,12].

Uterine MLA has a distinct immunophenotype [1,11]. Consequently, in this study, our hypothesis was that in uterine MLAs: (1) ER and PR expression would be either negative or focal positive, (2) p16 expression would be either negative or patchy, (3) p53 expression pattern would be wild-type, (4) *HER2* overexpression would be absent or rare, and (5) mesonephric markers, including GATA3, TTF1, and CD10, would be positively expressed in the majority of cases. While most of these tenets held true, we identified some cases where hormone receptor expression was intense and some cases where GATA3 expression was completely absent.

ER and PR are the most widely used IHC markers in the diagnosis of endometrial carcinoma. Reid-Nicholson et al. [37] reported that 83.8% (31/37) and 83.3% (35/42) of grade 1–2 ECs expressed ER and PR, respectively, whereas in SCs positive rates for ER and PR were both 54.2% (13/24). Shen et al. [38] reported a positive association between hormone receptor positivity and improved prognosis and responsiveness to hormonal treatment. We identified seven cases of uterine MLA that showed at least weak ER or PR expression in ≥5% of tumor cells. Six of these seven patients (85.7%) developed postoperative recurrence, and three (42.9%) experienced lung metastasis. More than half of these patients (4/7) had advanced-stage disease (one IIIA, two IIIC2, and one IVB). Among the 18 patients with hormone receptor-negative MLA, 66.7% (12/18) and 50% (9/18) developed postoperative recurrence and lung metastases, respectively. We did not observe any significant association between the expression status of GATA3, ER, PR, TTF1, and CD10 and FIGO stage, postoperative recurrence, lung metastasis, or DFS. Due to the small sample size in our study, it is unfortunately impossible to determine whether any significant association exists between hormone receptor expression and the prognosis of uterine MLA patients; however, hormone receptor-positive tumors do not appear to show significantly better outcome than those expressing hormone receptors. Further investigations using a larger cohort are warranted to compare the differences in clinicopathological features and prognosis between hormone receptor-positive and -negative uterine MLAs.

There have been several reports of hormone receptor positivity in uterine MLAs [11,39,40,41]. However, other studies have documented consistently negative results regarding hormone receptor expression in MLAs [1,4,5]. These contradictory findings complicate the understanding of MLA. Are MLAs Mullerian tumors with mesonephric-like differentiation (MLD), or true mesonephric tumors with Mullerian differentiation? Do two types of differently originated tumors exist with cross-differentiation, sharing the moniker mesonephric-like? While we cannot yet answer these questions, we encountered a case series of five endometrial ECs that exhibited MLD [42]. The cases demonstrated compactly aggregated small tubules and eosinophilic intraluminal secretions, both of which are the classic histological features of MLA. However, the presence of microscopic foci showing typical endometrioid histology, endometrioid premalignant lesions (i.e., atypical hyperplasia/endometrioid intraepithelial neoplasia), and either squamous or mucinous differentiation argued against the diagnosis of MLA. Furthermore, IHC revealed that these tumors were diffusely and strongly positive for hormone receptors, albeit with focal and strong GATA3 immunoreactivity. We therefore find it reasonable to assume that these cases are ECs with MLD. We were able to rule out the possibility of mixed EC and MLA because most of the tumor cells with GATA3 and PAX2 expression demonstrated moderate-to-strong nuclear immunoreactivity for ER and PR. Furthermore, the tumor cells expressing both hormone receptors and mesonephric markers were intermingled with those with only one of the two [42]. Our assumption is further strengthened by the molecular findings which showed that none of the tumors featured pathogenic *KRAS* mutation.

Conversely, in our study, a small subset of uterine MLAs unexpectedly showed positivity for hormone receptors. Four MLAs (16.0%) were positive for either ER or PR in ≥25% of tumor cells, with at least moderate staining intensity. The evident expression of hormonal receptors in these tumors raises the possibility that at least some MLAs exhibit endometrioid differentiation. After careful scrutiny, it is natural to conclude that while some MLAs may be Mullerian tumors with MLD [43], others are true mesonephric tumors with Mullerian differentiation.

Low-grade endometrial EC typically expresses both ER and PR and wild-type p53 IHC pattern and non-diffuse p16 positivity. In contrast, almost all SC exhibits aberrant p53 expression since it harbors pathogenic tumor protein 53 (*TP53*) mutation. Approximately two-thirds of endometrial SC cases show p53 overexpression, indicating a missense *TP53* mutation, and the remaining one-third display a complete absence of p53 staining, indicating a truncating *TP53* mutation [44]. In addition, SC typically shows diffuse and strong p16 immunoreactivity in conjunction with either negative expression or weak focal positivity for hormonal receptors. Similar to SC, a subset of high-grade EC (i.e., p53mut EC) harbors pathogenic *TP53* mutation and shows mutant p53 IHC pattern, diffuse p16 positivity, and/or lack of ER and PR expression. A panel of IHC with p16, p53, ER, and PR can be used to distinguish endometrial SC, p53mut EC, and low-grade EC from uterine MLA because the latter reacts uniformly with hormonal receptors and the former two express p16 and p53 aberrantly. In this study, no tumors showed diffuse p16 positivity, and none exhibited a mutant p53 IHC pattern. Consequently, if a case presents with mesonephric-like morphology but conflicting IHC results complicated by diffuse and strong p16 immunoreactivity, aberrant p53 expression, and/or pathogenic *TP53* mutation, the diagnosing pathologists should consider the possibility of endometrial SC or p53mut EC showing MLD [8]. If a case displays diffuse hormone receptor positivity with moderate-to-strong staining intensity, the diagnosis of endometrial low-grade EC showing MLD is favored [42].

*HER2* gene amplification is found in approximately 30% of endometrial SCs [45]. The frequency of HER2 positivity in MLA has not yet been reported. In this study, 20.0% (6/25) MLAs exhibited equivocal HER2 immunoreactivity. Adhering to the recommendations for HER2 testing in uterine tumors [31], we performed *HER2* FISH, confirming that none of the equivocal cases also exhibited *HER2* gene amplification. Although we did not find any MLA tumors with concomitant *HER2* gene amplification, further investigations are warranted to ascertain whether this is generally true of MLA or simply a result of our relatively small sample size.

The recently suggested diagnostic criteria for MLA are: (1) having characteristic morphology of MA or MLA, (2) positivity for at least one of the IHC markers, including GATA3, (3) negativity or focal positivity for ER, and (4) pathogenic *KRAS* mutation [7]. In this study, not all MLA tumors were positive for GATA3. Particularly, two tumors showed complete absence of GATA3 expression. Although GATA3 negativity argues against a mesonephric origin, the presence of the classic MLA morphological features, hormone receptor negativity, wild-type p53 expression pattern, patchy p16 positivity, and, most importantly, positivity for other mesonephric markers and a pathogenic *KRAS* mutation were all compatible with the diagnosis of MLA. We confirmed that the two GATA3-negative tumors showed positive expression for TTF1 and CD10, negative hormone receptor expression, wild-type p53 IHC patterns, and patchy p16, and exhibited pathogenic *KRAS* mutations.

GATA3 recognizes the nucleotide sequences of G-A-T-A and regulates differentiation of many different cell types [46]. While its expression is widely acknowledged in the mammary and urothelial epithelium and T lymphocytes, GATA3 is one of the most important markers for the diagnosis of benign and malignant mesonephric lesions [14,35,46]. However, GATA3 expression sensitivity is somewhat controversial across different studies: one study reported that the sensitivity and specificity of GATA3 in the cervical mesonephric lesions are as high as greater than 90%, although the sensitivity and specificity are lower in the mesonephric lesions near the adnexa [14]. A recent meta-analysis of MAs and MLAs showed <50% GATA3 positivity [36]. Since GATA3 positivity is not universal across all MA and MLAs, the selection of an optimal antibody and IHC method could be important for this ancillary test to be maximally utilized. We used the L50-823 clone (Ventana Medical Systems, Roche, Oro Valley, AZ, USA) for GATA3 antibody, which is commonly used in studies on MA and MLA [7,12,43]. Exploration of different GATA3 antibodies in uterine MLAs as well as other histological subtypes of endometrial carcinoma would be valuable.

This study confirmed previous data shown by Pors et al. [12] and Euscher et al. [5] on the expression of GATA3 and TTF1 in uterine MLA. The majority of cases were positive for GATA3 and TTF1, even though both markers tended to be stained focal in approximately half of cases. We found that all our uterine MLAs expressed at least one of the two markers, and that the mean H-score of GATA3 (116.2) and TTF1 (111.9) was similar. The inverse staining pattern of GATA3 and TTF1 in eight cases in our series is in agreement with that of the aforementioned previous studies [5,12]. We noted four cases in which GATA3 was expressed very focally (5–10%) but the TTF1 staining was strongly positive, and the other four cases in which GATA3 showed much stronger expression than TTF1. The use of both GATA3 and TTF1 would facilitate arriving at a diagnosis of MLA correctly, especially in small biopsy samples [5,12]. In addition, we observed that TTF1 immunostaining was geographically heterogeneous in more than half (57.1%). There was no significant difference in histological features between the areas showing a complete lack of TTF1 staining and the adjacent tumor cell clusters that were strongly positive for TTF1. The mechanisms leading to this intratumoral heterogeneity of TTF1 expression in uterine MLA are unknown.

In 2013, The Cancer Genome Atlas (TCGA) published a comprehensive genomic analysis of endometrial carcinoma [47,48]. The TCGA classified endometrial carcinoma cases into four prognostically distinct subgroups, including (1) ultramutated; (2) hypermutated; (3) copy number-low; and (4) copy number-high. In 2015, the Proactive Molecular Risk Classifier for Endometrial Cancer (ProMisE) was developed to recapitulate the four TCGA subgroups by using IHC for p53 and mismatch repair (MMR) proteins, along with sequencing for DNA polymerase ε (*POLE*) exonuclease domain mutation (EDM) [49]. Four subgroups of the ProMisE, serving as corollaries to the TGCA subgroups described above, are (1) *POLE*-mutated; (2) MMR-deficient; (3) no specific molecular profile (NSMP); and (4) p53-mutated. At a molecular level, most MLAs harbor activating *KRAS* mutations [1,6,11,13,15,35]. A few available data have suggested that uterine MLA is not associated with either a *POLE* EDM or MMR-deficient signature [6,50]. Since uterine MLAs almost never exhibit *TP53* mutation or microsatellite instability [11], it seems that MLA falls into the NSMP subgroup. However, since the biological behavior of MLA is consistently described as aggressive [7], the inclusion of this entity into the high-risk non-endometrioid group would be more appropriate [50].

Clinicopathological characteristics of uterine MLCS have rarely been documented, since only a few cases of MLCS have been reported in the uterus and ovary [12,51,52,53,54,55]. We described four cases of uterine MLCS. They showed definitive morphological and immunohistochemical evidence of biphasic tumors comprising malignant epithelial and mesenchymal components. In this study, MLA was the only epithelial component of all MLCS cases, occupying more than half of the entire tumor volume. The mesenchymal component was high-grade nonspecific sarcoma without exhibiting smooth muscle or endometrial stromal differentiation. We observed no heterologous mesenchymal component. Similar to our observation, Meguro et al. [54] did not find any heterologous component in their case of cervical MLCS. In contrast, Pors et al. [12] demonstrated heterologous chondrosarcomatous components in two of three cervical MLCS cases, and d’Amati et al. [51] also reported that a part (5%) of the ovarian MLCS consisted of a chondrosarcomatous element. They stated that this heterologous element along with MLA metastasized to the pericolic fat of the intestine, whereas the omental and peritoneal metastatic nodules revealed MLA only. The latter finding was in line with that of this study, in which nodal and distant metastatic lesions consisted exclusively of MLA without mesenchymal component in three MLCS cases. A previous study on uterine conventional carcinosarcoma showed that the epithelial component determined the prognosis of patients by causing the majority of metastases and vascular invasion [56]. Further investigations are warranted to examine the clinicopathological significance of each component in uterine MLCS. In addition, we noted that some tumor cells in the mesenchymal component displayed weak immunoreactivity for EMA and CK7, accompanied with a significant reduction or loss of expression of those epithelial markers in the adjacent epithelial component. Given the fact that uterine conventional carcinosarcoma is currently considered as a metaplastic high-grade endometrial carcinoma [57,58], our findings raise the possibility that MLCS may be also a metaplastic MLA in which the mesenchymal component retains at least partially the morphological and/or immunohistochemical features of MLA (i.e., the mesenchymal morphology of MLCS may represent a result of epithelial–mesenchymal transition or transdifferentiation of MLA). Further studies are necessary to investigate whether uterine MLCS shows similar immunohistochemical and molecular features to those of MLA and to confirm the monoclonal origin of uterine MLCS.

## 5. Conclusions

We analyzed the expression of the selected eight IHC markers in 25 cases of MLA. A small subset of uterine MLAs displayed atypical immunophenotypes, particularly (1) focal but strong expression of ER and/or PR, (2) a complete absence of GATA3 immunoreactivity, and (3) the co-expression of mesonephric markers and hormone receptors. These unusual IHC results complicate the differential diagnosis of MLA. On the other hand, all cases uniformly showed patchy p16 positivity and a wild-type p53 IHC pattern. An inverse staining pattern of GATA3 and TTF1 was noted in several MLA cases. We suggest that a panel of several IHC markers must be used in the differential diagnosis of uterine carcinoma showing mesonephric-like morphology.

## Figures and Tables

**Table 1:** Antibodies used.

Antibody	Clone	Company	Dilution
GATA3	L50-823	Ventana Medical Systems (Roche, Oro Valley, AZ, USA)	Prediluted
TTF1	8G7G3/1	Dako (Agilent Technologies, Santa Clara, CA, USA)	1:100
CD10	56C6	Novocastra (Leica Biosystems, Buffalo Grove, IL, USA)	1:100
ER	6F11	Novocastra (Leica Biosystems, Buffalo Grove, IL, USA)	1:200
PR	16	Novocastra (Leica Biosystems, Buffalo Grove, IL, USA)	1:800
p53	DO-7	Novocastra (Leica Biosystems, Buffalo Grove, IL, USA)	1:200
p16	E6H4	Ventana Medical Systems (Roche, Oro Valley, AZ, USA)	Prediluted
HER2	4B5	Ventana Medical Systems (Roche, Oro Valley, AZ, USA)	Prediluted

**Table 2:** Clinicopathological characteristics.

Case No.	Age (Years)	Initial FIGO Stage	Recurrence	Lung Metastasis	DFS (Months)
1	50	IA	Yes	Yes	53
2	62	IA	Yes	Yes	60
3	62	IIIC2	Yes	Yes	33
4	53	IIIA	Yes	No	55
5	67	IIIC2	Yes	No	12
6	47	IVB	Yes	No	5
7	59	IIIC1	NA (recent)	NA (recent)	NA (recent)
8	48	IIIC1	Yes	No	20
9	72	IA	Yes	Yes	22
10	58	IIIB	No	No	19
11	59	IIIC1	Yes	Yes	15
12	52	IIIB	No	No	15
13	56	IIIB	No	No	12
14	66	IB	No	No	11
15	68	IVB	Yes	Yes	8
16	57	IVB	Yes	No	4
17	77	IA	NA (recent)	NA (recent)	NA (recent)
18	55	II	Yes	Yes	26
19	69	IVB	No	Yes	36
20	61	IB	Yes	Yes	5
21	62	IVB	Yes	Yes	16
22	43	II	Yes	Yes	39
23	46	IIIC1	Yes	Yes	6
24	60	IB	Yes	Yes	15
25	61	IVB	Yes	Yes	13

**Abbreviations:** NA, not applicable; DFS, disease-free survival.

**Table 3:** IHC and NGS results.

Case No.	GATA3					ER					PR					p16	p53	TTF1					CD10					HER2 IHC	*HER2* FISH	*KRAS*
	S (%)	M (%)	W (%)	N (%)	H	S (%)	M (%)	W (%)	N (%)	H	S (%)	M (%)	W (%)	N (%)	H			S (%)	M (%)	W (%)	N (%)	H	S (%)	M (%)	W (%)	N (%)	H			
1	0	40	30	30	110	0	0	0	100	0	10	30	0	60	90	WT	P	0	5	0	95	10	5	5	0	90	25	1+		p.G12D
2	0	5	5	90	15	0	0	1	99	1	0	0	1	99	1	WT	P	0	5	0	95	10	0	0	0	100	0	0		WT
3	5	5	0	90	25	10	20	10	60	80	0	0	0	100	0	WT	P	80	0	0	20	240	10	0	0	90	30	0		p.G12D
4	80	10	0	10	260	10	20	20	50	90	0	0	0	100	0	WT	P	0	40	30	30	110	20	0	0	80	60	1+		WT
5	80	10	0	10	260	0	10	10	80	30	0	0	0	100	0	WT	N	80	0	0	20	240	50	0	0	50	150	1+		p.G12D
6	10	20	20	50	90	0	0	5	95	5	0	0	0	100	0	WT	P	NA	NA	NA	NA	NA	NA	NA	NA	NA	NA	0		NA
7	0	5	5	90	15	0	0	0	100	0	0	0	0	100	0	WT	P	30	20	0	50	130	5	0	0	95	15	0		p.G12V
8	10	30	0	60	90	0	0	0	100	0	0	0	0	100	0	WT	N	60	0	0	40	180	30	20	0	50	130	0		p.G12D
9	0	5	0	95	10	0	1	4	95	6	0	0	0	100	0	WT	P	100	0	0	0	300	50	0	0	50	150	2+	N	p.G12V
10	15	60	10	15	175	0	0	0	100	0	0	0	0	100	0	WT	P	50	0	0	50	150	30	20	0	50	130	0		p.G12D
11	20	20	0	60	100	0	0	0	100	0	0	0	0	100	0	WT	P	40	0	0	60	120	20	20	0	60	100	1+		p.G12C
12	20	60	10	10	190	0	0	0	100	0	0	0	0	100	0	WT	P	0	30	0	70	60	0	0	0	100	0	1+		WT
13	1	2	2	95	9	0	0	1	99	1	0	0	0	100	0	WT	P	0	0	10	90	10	50	10	0	40	170	0		WT
14	90	5	0	5	280	15	10	0	75	65	4	1	0	95	14	WT	P	5	0	0	95	15	0	0	0	100	0	2+	N	WT
15	10	20	50	20	120	0	0	0	100	0	0	0	0	100	0	WT	P	100	0	0	0	300	100	0	0	0	300	1+		p.G12D
16	80	5	0	15	250	0	0	0	100	0	0	0	0	100	0	WT	P	0	25	0	75	40	60	10	0	50	200	1+		p.G12V
17	10	15	15	60	75	0	0	0	100	0	0	0	0	100	0	WT	P	100	0	0	0	300	50	20	0	30	190	1+		p.G12V
18	0	0	0	100	0	0	0	0	100	0	0	0	0	100	0	WT	P	0	0	10	90	10	90	0	0	10	270	2+	N	p.G12V
19	100	0	0	0	300	0	0	0	100	0	0	0	0	100	0	WT	P	0	0	5	95	5	10	10	0	80	50	0		p.G12D
20	0	5	5	90	15	0	0	0	100	0	0	0	0	100	0	WT	P	20	20	0	60	100	5	0	0	95	15	2+	N	p.G12V
21	90	5	5	0	285	0	0	0	100	0	0	0	0	100	0	WT	P	0	0	10	90	10	0	0	0	100	0	2+	N	p.G13D
22	0	0	0	100	0	0	0	0	100	0	0	0	0	100	0	WT	P	0	5	0	95	10	20	10	0	70	80	0		p.G12D
23	10	20	10	60	80	0	0	0	100	0	0	0	0	100	0	WT	P	NA	NA	NA	NA	NA	NA	NA	NA	NA	NA	0		NA
24	10	20	0	70	70	0	0	0	100	0	0	0	0	100	0	WT	P	NA	NA	NA	NA	NA	NA	NA	NA	NA	NA	1+		NA
25	15	10	15	60	80	0	0	0	100	0	0	0	0	100	0	WT	P	NA	NA	NA	NA	NA	NA	NA	NA	NA	NA	0		NA

**Abbreviations:** H, histo score; M, moderate; N, negative; NA, not applicable; P, patchy; S, strong; W, weak; WT, wild-type.

**Table 4:** Summary of IHC results.

Antibody	Intensity	Proportion (%)	Patients (%)
GATA3	Moderate-to-strong	≥75	8 (32.0)
	Moderate-to-strong	25–49	9 (36.0)
	Negative	≥90	8 (32.0)
ER	Moderate-to-strong	25–49	3 (12.0)
	Weak-to-moderate	5–24	3 (12.0)
	Negative	≥99	19 (76.0)
PR	Moderate-to-strong	5–49	2 (8.0)
	Negative	≥99	23 (88.0)
p53	Wild-type		25 (100.0)
p16	Patchy		23 (92.0)
	Negative		2 (8.0)
TTF1	Moderate-to-strong	≥50	8 (32.0)
	Moderate-to-strong	25–49	5 (20.0)
	Negative	≥90	8 (32.0)
	Not applicable		4 (16.0)
CD10	Moderate-to-strong	≥50	9 (36.0)
	Moderate-to-strong	5–49	8 (32.0)
	Negative	≥99	4 (20.0)
	Not applicable		4 (16.0)
HER2	Negative (0–1+)		20 (80.0)
	Equivocal (2+)		5 (20.0)

**Table 5:** Associations of histo score for GATA3, ER, or PR with clinicopathological parameters, and DFS.

Parameter		Total (%)	GATA3			ER			PR		
			H < 50	H ≥50	*p* Value	H < 5	H ≥ 5	*p* Value	H < 5	H ≥ 5	*p* Value
Initial FIGO stage	I	7 (28.0)	3 (42.9)	4 (57.1)	0.640	5 (71.4)	2 (28.6)	1.000	5 (71.4)	2 (28.6)	0.070
	II–IV	18 (72.0)	5 (27.8)	13 (72.2)		14 (77.8)	4 (22.2)		18 (100.0)	0 (0.0)	
Recurrence	No	5 (20.0)	1 (20.0)	4 (80.0)	1.000	4 (80.0)	1 (20.0)	1.000	4 (80.0)	1 (20.0)	0.395
	Yes	18 (72.0)	6 (33.3)	12 (66.7)		13 (72.2)	5 (27.8)		17 (94.4)	1 (5.6)	
	NA	2 (8.0)									
Lung metastasis	No	9 (36.0)	1 (11.1)	8 (88.9)	0.176	5 (55.6)	4 (44.4)	0.162	8 (88.9)	1 (11.1)	1.000
	Yes	14 (56.0)	6 (42.9)	8 (57.1)		12 (85.7)	2 (14.3)		13 (92.9)	1 (7.1)	
	NA	2 (8.0)									
Mean DFS (months)			31.6	22.8	0.237	24.8	26.3	0.972	23.4	53.0	0.384

**Abbreviations:** H, histo score; NA, not applicable; DFS, disease-free survival.

**Table 6:** Associations of histo score for TTF1 or CD10 with clinicopathological parameters, and DFS.

Parameter		Total (%)	TTF1			CD10		
			H < 50	H ≥ 50	*p* Value	H < 50	H ≥ 50	*p* Value
Initial FIGO stage	I	6 (28.6)	3 (50.0)	3 (50.0)	1.000	4 (66.7)	2 (33.3)	0.146
	II–IV	15 (71.4)	6 (40.0)	9 (60.0)		4 (26.7)	11 (73.3)	
Recurrence	No	5 (23.8)	3 (60.0)	2 (40.0)	0.628	2 (40.0)	3 (60.0)	1.000
	Yes	14 (66.7)	6 (42.9)	8 (57.1)		5 (35.7)	9 (64.3)	
	NA	2 (9.5)						
Lung metastasis	No	8 (38.1)	3 (37.5)	5 (62.5)	0.650	2 (25.0)	6 (75.0)	0.633
	Yes	11 (52.4)	6 (54.5)	5 (45.5)		5 (45.5)	6 (54.5)	
	NA	2 (9.5)						
Mean DFS (months)			34.5	23.5	0.233	35.4	25.1	0.297

**Abbreviations:** H, histo score; NA, not applicable; DFS, disease-free survival.
